# Serum free culture for the expansion and study of type 2 innate lymphoid cells

**DOI:** 10.1038/s41598-021-91500-z

**Published:** 2021-06-10

**Authors:** Pablo de Lucía Finkel, Christopher Sherwood, Iryna Saranchova, Wenjing Xia, Lonna Munro, Cheryl G. Pfeifer, James M. Piret, Wilfred A. Jefferies

**Affiliations:** 1 Michael Smith Laboratories, University of British Columbia, 2185 East Mall, Vancouver, BC V6T 1Z4 Canada; 2 The Vancouver Prostate Centre, Vancouver General Hospital, 2660 Oak Street, Vancouver, BC V6H 3Z6 Canada; 3 Department of Microbiology and Immunology, University of British Columbia, 2350 Health Sciences Mall, Vancouver, BC V6T 1Z4 Canada; 4 Centre for Blood Research, University of British Columbia, 2350 Health Sciences Mall, Vancouver, BC V6T 1Z4 Canada; 5 The Djavad Mowafaghian Centre for Brain Health, University of British Columbia, 2215 Wesbrook Mall, Vancouver, BC V6T 1Z4 Canada; 6 Department of Medical Genetics, University of British Columbia, 2350 Health Sciences Mall, Vancouver, BC V6T 1Z4 Canada; 7 Department of Zoology, University of British Columbia, 6270 University Blvd, Vancouver, BC V6T 1Z4 Canada; 8 Department of Urologic Sciences, University of British Columbia, Vancouver, BC V5Z 1M9 Canada; 9 Department of Chemical and Biological Engineering, University of British Columbia, 2360 East Mall, Vancouver, BC V6T 1Z3 Canada; 10 School of Biomedical Engineering, The University of British Columbia, 2222 Health Sciences Mall, Vancouver, BC V6T 1Z3 Canada

**Keywords:** Isolation, separation and purification, Microscopy, Cell culture, Cell growth, Cell division, Biological techniques

## Abstract

Type 2 innate lymphoid cells (ILC2s) were discovered approximately ten years ago and their clinical relevance is gaining greater importance. However, their successful isolation from mammalian tissues and in vitro culture and expansion continues to pose challenges. This is partly due to their scarcity compared to other leukocyte populations, but also because our current knowledge of ILC2 biology is incomplete. This study is focused on ST2^+^ IL-25R^lo^ lung resident ILC2s and demonstrate for the first time a methodology allowing mouse type 2 innate lymphoid cells to be cultured, and their numbers expanded in serum-free medium supplemented with Interleukins IL-33, IL-2, IL-7 and TSLP. The procedures described methods to isolate ILC2s and support their growth for up to a week while maintaining their phenotype. During this time, they significantly expand from low to high cell concentrations. Furthermore, for the first time, sub-cultures of primary ILC2 purifications in larger 24- and 6-well plates were undertaken in order to compare their growth in other media. In culture, ILC2s had doubling times of 21 h, a growth rate of 0.032 h^−1^ and could be sub-cultured in early or late phases of exponential growth. These studies form the basis for expanding ILC2 populations that will facilitate the study and potential applications of these rare cells under defined, serum-free conditions.

## Introduction

Innate lymphocytes (ILCs) are the innate equivalents of T lymphocytes. Based on their function and protein expression, they can be grouped into three different categories of ILCs: types 1, 2 and 3^1^. Type-2 innate lymphoid cell (ILC2) are rare cells that are known as type 2 cytokine secretors in response to helminthic infections, but also as major drivers of allergen sensitization and allergic lung inflammation^2^. ILC2 engagement is initiated at mucosal sites by epithelial cell—derived alarmins: thymic stromal lymphopoietin (TSLP) and interleukins (IL) IL-25 and IL-33^3,4^.

Lacking adaptive antigen receptors, ILC2s sense the microenvironment via cytokine receptors, and in response, regulate immunity by secretion of a large number of cytokines^5–11^. IL-33 not only activates ILC2s, but also promotes eggress of newly generated ILC2s from the bone marrow to the lungs^10,12–14^ and leads to systemic ST2^+^ (IL-33R) ILC2 expansion^15^. Also, ILC2s modulate adaptive immune responses by cell-to-cell contact through the expression of MHC-II molecules^5,16^.

ILC2s originate from Lin^−^ CD127^+^ Flt3^+^ common lymphoid progenitors in the bone marrow that can be isolated and induced to differentiate into ILC2s via IL-33, IL-7 and Notch signaling^17,18^. ILC2s are generally tissue resident cells found in the lungs, adipose tissues, mesenteric lymph nodes, intestine, liver, spleen, blood and meninges^19–23^. The most common source for “conventional” ILC2s is the lungs^7,24–27^. These cells express the transcription factor GATA-3 and in its absence the development and function of ILC2s is impaired^1,28–30^. In a departure from the existing dogma, the function of ILC2s in T_H_1 responses against tumours was recently discovered^31^. The data supports a new paradigm for cancer immunology, where the IL-33/ILC2 axis enhances additional T_H_1 effector function that assists and mediates anti-cancer CTL immune responses and reduces tumour metastasis. Thus, ILC2s may have important therapeutic utility amongst the multitude of emerging cancer immunotherapies and this motivated our studies on how to expand ILC2s in culture.

ILC2s are a challenging cell population to explore experimentally due to their scarcity^32^. In mice, ILC2s represent 0.25–1% of the total leukocyte population in the lungs^33^, which appears to be a depot for these cells, as their numbers in the lungs decrease after peripheral immune-activation. Female mice have been reported to harbour greater ILC2 numbers than males potentially due to a lack of the maturity marker KLRG1, which inhibits ILC2 function upon binding to E-cadherin^34^. Intraperitoneal or intranasal IL-33 pre-treatment has proven to increase ILC2 frequency in the lungs to, thereby, increase yields during isolations^24,35,36^. An ILC2 isolation yields approximately 1,000 cells per mouse, whereas from alarmin pre-treated mice it is possible to obtain 150,000–1,000,000 cells per animal (dose dependent). In general, it has proven difficult to expand primary ILC2s in vitro, where sub-optimal medium conditions or inadequate cytokine combinations can easily cause cell viability to plummet and limit their longevity in culture^37^. Furthermore, given the potential for ILC2-based cell therapies, replacing fetal bovine serum (FBS) media with serum-free culture media is desirable in order to increase cell production consistency and quality assurance (reduction of reactive components) in a clinical context^38–40^.

Here, we isolated ST2^+^ IL-25R^lo^ lung ILC2s and expanded them in serum-free medium containing IL-33, TSLP, IL-2 and IL-7. This is an alternative to RPMI 1640 containing 10% FBS (RPMI/FBS) supplemented with ILC2-stimulating cytokines, which is the most common culture media for ILC2s^2,31,37,41^. This culture methodology will enable future research and applications for ILC2s in adoptive transfer approaches and other settings.

## Materials and methods

Relevant guidelines and regulations were followed for the live animal experiments; all protocols and procedures involving the care and use of animals in these studies were reviewed and approved by the Animal Care Committee of the University of British Columbia; which is governed by the Canadian Council of Animal Care. All studies involving live animals complied with ARRIVE guidelines^42^.

### Tissue processing and ILC2 isolation

Lungs from mice were harvested and cut into small pieces using a razor blade and digested for 30 min at 37 °C in a shaker platform (200 rpm) in 10 mL of digestion medium per 5 pairs of lungs. The digestion medium contained RPMI 1640 (Gibco #11875-093) with 100 U penicillin and streptomycin (P/S) (Invitrogen #15140-122) and 10% FBS (Gibco #A3840302), as well as 1 mL collagenase/hyaluronidase (StemCell #07912) and 1.5 mL DNase I (StemCell #07900) per 5 pairs of lungs.

The digested pieces of lung tissue were placed onto a 70 µm cell strainer. Using the plunger end of a 3 mL syringe, the tissue was mashed through the strainer and rinsed with 5 mL RPMI 1640 to a total of 15 mL. Cells were centrifuged for 6 min at 1600-1700 rpm. The supernatant was carefully removed and the pellet re-suspended in 20 mL ammonium chloride solution (StemCell #07,800), then incubated at room temperature for 5 min to lyse the erythrocytes. After neutralization with 30 mL of FACS buffer cells were counted and washed (6 min, 1600–1700 rpm) in a total volume of 50 mL (full 50 mL Falcon Tube). FACS buffer was made of DPBS (Gibco #14190-136) with 2% FBS.

After counting cells, they were re-suspended in the appropriate volume of FACS buffer to obtain 1 × 10^8^ cells/mL, then enriched for ILC2s using an EasySep Mouse ILC2 Enrichment Kit (StemCell #19842), which reduces sorting time and increases ILC2 recovery both from naïve and IL-33–treated lungs. Prior to sorting, the cells were stained with FITC-conjugated lineage marker mouse antibodies purchased from Invitrogen (CD3ε/γ #11-0032-80, CD4 #11-042-81, CD8α #11-0081-81, CD19 #11-0193-81, TCRβ #11-5961-81, NK1.1 #11-5941-81, TER119 #11-5921-81, CD11c #11-0114-81, CD11b #11-0112-41 and Ly-6G/C #11-9668-80) and the ILC2 markers purchased from BioLegend: PE-conjugated CD127 (IL-7 receptor, #135009), PerCP-Cy5.5-conjugated ST2 (IL-33 receptor #145312), BV605-conjugated Thy1.2 (#140317) and BV421-conjugated CD45 (#103133). ILC2s were then phenotypically assessed and sorted using a BD FACS Aria II machine to purify the population.

### Mouse ILC2 in vitro culture and analysis

C57Bl/6 J wild type mice (Jackson Laboratory strain 000664) were pre-treated with 600 ng of intraperitoneal injection of mouse recombinant IL-33 (R&D Systems #3626-ML-010) one week prior to isolation injecting every other day for a total of three days (one 200 ng injection each day).

For optimal growth ILC2s were cultured in serum-free StemSpan SFEM II (StemCell #09655) supplemented with 100 ng/mL of recombinant mouse IL-33 (Invitrogen #14-8332-80) and TSLP (Invitrogen#14-8498-90) for ILC2 activation, 25 ng/mL of rmIL-7 to promote survival (R&D Systems #407-ML-005) and 1000 Units/mL of recombinant human IL-2 to enhance proliferation (StemCell #78036.3). The media also contained P/S.

For experimental purposes, ILC2s were also cultured in RPMI 1640 with 10% FBS supplemented with the same cytokines and antibiotics. In both scenarios cells were kept at 37 °C during experiments. Cells were cultured in U-bottom, non-treated 96- or flat bottom 24-well plates. Fed-batch wells had half of their volume of media changed every other day and 50% of the wells were triturated with a pipette to avoid cell clumps in all experiments.

Other culture conditions with IMDM (Isocove’s MDM) plus FBS or DMEM plus FCS could be compared in the future that more closely parallel the media in serum-free StemSpan SFEM II.

ILC2s in culture were counted every day using a Cedex HiRes automated cell counter. Prior to loading a 300µL sample into the Cedex Sample Cups, cultures were pipetted to disaggregate cell clumps. Samples were usually diluted with fresh media to 1:2 ratios to facilitate cell counting. The precision level was set to *superior*, which allows for approximately 8 images for analysis per sample and the cell type was set as *lymphocyte*, which is the minimum size and appropriate for analysis of cells with a diameter of about 6 µm, such as ILC2s.

Glucose and lactate levels were measured every day by analyzing 90µL of ILC2 culture supernatant using a Stat Profile pHOx Ultra Bloodgas Analyzer from Nova Biomedical. Photos of the ILC2 culture in StemSpan were taken using a Canon EOS DSLR camera attached to a Motic confocal microscope. Images were processed using the software Camera Control Pro from Nikon and ImageJ v1.8.0. Graphical figures were produced in GraphPad Prism v8.0.0.

### Calculation of ILC2 growth rate and doubling time

The average cell growth rate was calculated based on the exponential phase of three different ILC2 cultures in 1 mL of media in a 24-well plate. The equation employed is the following:

$$ C_{f} = C_{i} \times e^{{k\left( {t_{f} - t_{i} } \right)}} $$

where $k$ represents the cellular mean growth constant or growth rate, $C_{i}$ the initial cell number and $C_{f}$ the final cell number at the end of the exponential phase. Initial time (h) is depicted by $t_{i}$ and represents the beginning of the culture, whereas $t_{f}$ is the time that has passed at the end of the exponential phase. Doubling time $\left( {t_{\frac{2}{1}} } \right)$ was calculated with the following equation:

$$ t_{\frac{2}{1}} = \frac{ln2}{k}. $$

### Cytokine production assays

The secretion of IL-5 and IL-13 was assessed by enzyme-linked immunosorbent assays (ELISA) according to the manufacturer protocol using as standards recombinant murine IL-5 (PeproTech #215-15) and recombinant murine IL-13 (PeproTech #210-13).

## Results

### Mouse ILC2s can be expanded in serum-free medium

**Figure 1:**
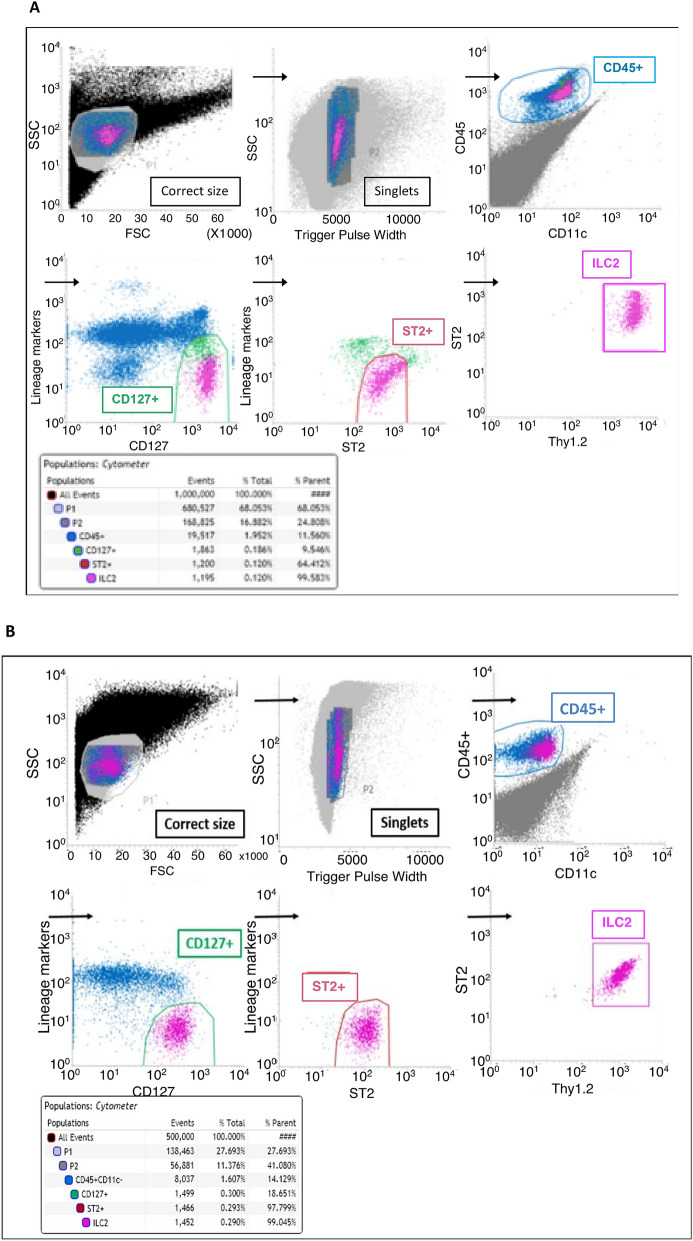
Gating strategy for ILC2s from lungs were sorted by FACS as Lin^−^ ST2^+^ CD127^+^ CD90.2^+^ cells. (**A**) ILC2s isolated from untreated animals (0.1% of total). Sequential gating strategy was based on cell size [small and non-granular cells, forward scatter (FSC) versus side scatter (SSC)], depletion of doublets (FSC vs. Trigger Pulse Width), and selection of CD45 + cells. Cells with lineage-related markers were rigorously depleted during isolation process and the resultant cell numbers at each stage of purification is shown. (**B**) ILC2s isolated from IL-33-treated animals (0.3% of total). The final gate (double positive for ST2 and Thy1.2) includes ILC2s isolated and sorted from lung tissue. Purified Lin^−^ ST2^+^ CD127^+^ CD90.2^+^ ILC2s express GATA3, IL5 and IL13 and this is stable during culturing.

**Figure 2:**
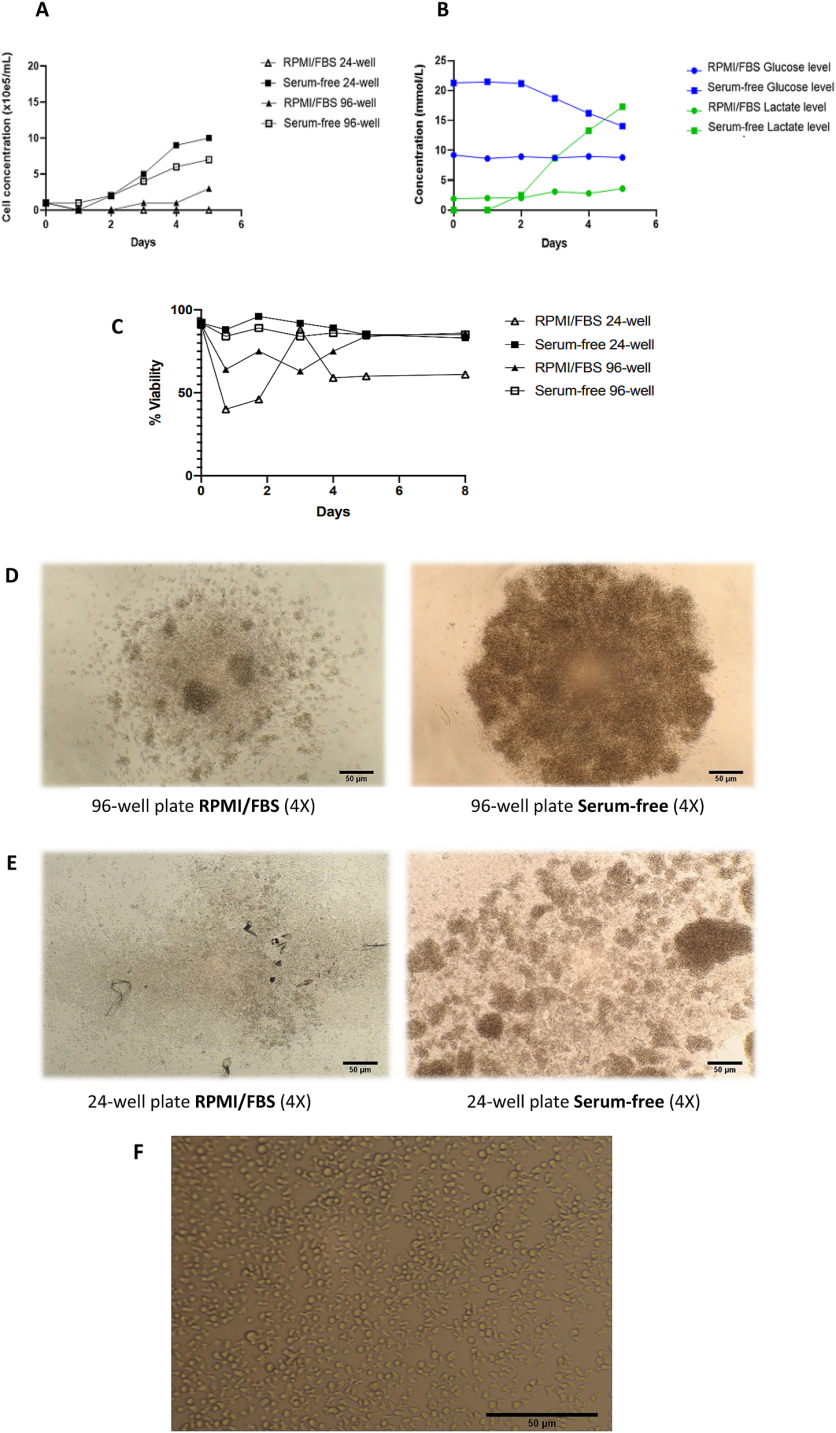
Serum-free medium supplemented with IL-33, TSLP, IL-2 and IL-7 promotes mouse ILC2 growth and expansion more than RPMI 1640 with 10% fetal bovine serum. (**A**) Starting at the same cell concentration, serum-free medium promotes culture expansion significantly more than RPMI/FBS in both round bottom 96- and flat bottom 24-well plates. (**B**) This difference in growth is also reflected in terms of glucose consumption and lactate production. (**C**) ILC2s presented high and consistent viabilities in serum-free medium as opposed to RPMI/FBS cultures. (**D**–**E**) Cell density is also clearly different between the media in the (**D**) round bottom 96- and (**E**) flat bottom 24-well plates. (**F**) Representative image of healthy ILC2 morphology in a 24-well plate (10 × magnification, serum-free medium). Immediately after harvest, cells are small and round. This morphology is maintained in RPMI/FBS whereas in serum-free medium they become irregular.

In mice, ILC2s were harvested from the lungs and purified by FACS as Lin ST2^+^CD127^+^CD90.2^+^ cells in the gating strategy described in Fig. 1A,B and then cultured in 24-well flat bottomed and 96-well round bottomed plates both in RPMI/FBS and serum-free medium containing IL-33, TSLP, IL-2 and IL-7. Having previously tested a number of different combination, we found that IL-33, TSLP, IL-2 and IL-7 gave the best propagation over any other combination we studied. The CD127 antibody blocks IL-7R, resulting in ILC2s which are unable to be stimulated by IL-7 in the ex vivo culture, and may affect the growth of ILC2s. As shown in Fig. 2A, the cell number has been increased tenfold more in the serum-free medium, when compared to the RPMI/FBS, reaching over 1 × 10^6^ viable cells/mL in 5 days from an initial explant of 1 × 10^5^ cells/mL in the 24-well plate. In the 96-well plate, however, ILC2s in serum-free medium increased 3.5-fold more than in RPMI/FBS, which highlights the appropriateness of larger culture spaces when employing serum-free medium. To further support our findings, we measured the glucose consumption and subsequent lactate production in every culture, and the wells containing serum-free media displayed greater glucose uptake and lactate production (Fig. 2B). The percentage (%) of ILC2 viabilities per day are summarized in Fig. 2C, where cells in serum-free medium kept a consistent viability (> 90%) throughout the 8 days, as opposed to the highly variable and reduced viability of ILC2s in RPMI/FBS cultures. The cell count differences are also illustrated by the morphologies seen in Figs. 2D,E. The cell morphology immediately after harvest in the serum-free medium is shown in Fig. 2F. Immediately after harvest, cells are small and round. This morphology is maintained in RPMI/FBS (not shown) whereas in serum-free media they become irregular and acquire a water drop-like shape over time.

We have also calculated the average ILC2 growth rate from three different cultures in serum-free medium in a 24-well plate. The growth constant *µ* as the number of cell generations per unit time, and in mouse ILC2s it turns out to be 0.032 generations per hour, or 0.032 h^−1^. This value is of importance to calculate the doubling time for ILC2s, which was determined to be 21 h to double the size of the population in serum-free medium. In RPMI/FBS, however, ILC2s grow more slowly and have a doubling time of almost 70 h; greater than 3 times longer.

**Figure 3:**
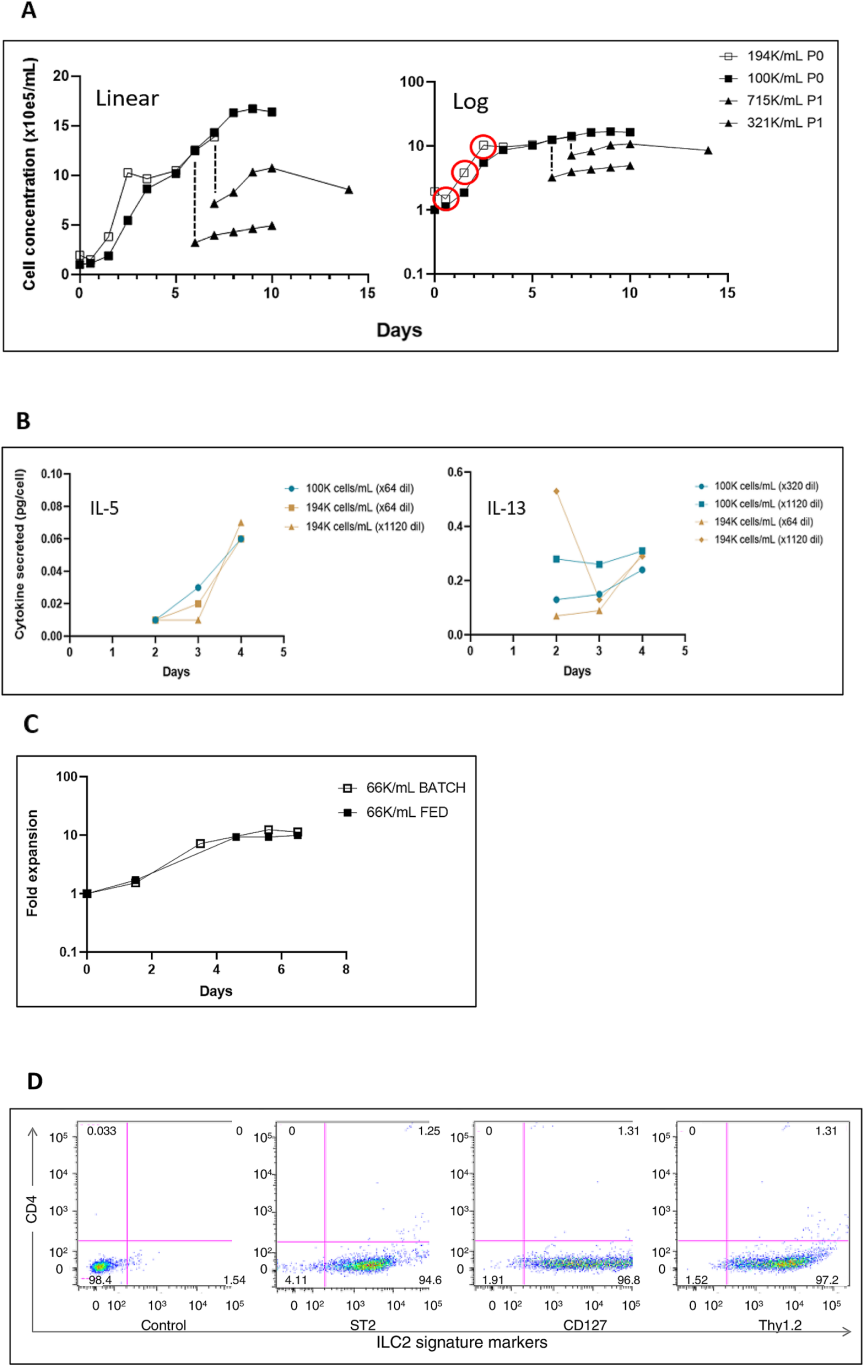
Subcultures of Primary ILC2s using serum-free medium supplemented with IL-33, TSLP, IL-2 and IL-7. (**A**) The starting cell concentration of the original explant (P0) does not have a significant effect on expansion. The three time points highlighted in red represent the exponential phase of cellular growth in the linear and log version of the same growth curve. ILC2 growth rates were calculated during these periods of exponential expansion. ILC2 sub-cultures (P1) at different concentrations seeded on days 6 and 7 after the exponential phase are viable, grow slowly and cells start dying after five days. (**B**) Amount of IL-5 and IL-13 secreted per ILC2 individual cell based on Fig. 3A. We have included two different initial cell concentrations that have been diluted differently in an ELISA assay (not included). (**C**) ILC2 original explants seeded at lower concentrations experience a significant fold expansion in less than 7 days. In addition, changing culture medium every other day (FED) does not have a noticeable impact in expansion compared to batch cultures, where the medium was never changed. (**D**) Cells cultured serum free-media continue to express ILC2-signature-markers after two weeks in culture.

We then seeded both 1 × 10^5^ and 1.94 × 10^5^ ILC2s/mL in a 24-well plate and observed a somewhat shorter lag at the higher inoculum concentration with a both displaying similar growth pattern (Fig. 3A). Moreover, we also sub-cultured the 10^5^ cells/mL explant (P0) to two different cell concentrations (P1) on days 6 and 7. These ILC2 remained viable and expanded slowly but viable cell concentration decreased five days later (Fig. 3A). We confirmed functionality of the culture-expanded cells by measuring cell IL-5 and IL-13 production (Fig. 3B) and by assessing their function in adoptive transfer assays (de Lucía Finkel et al., submitted).

Finally, in a 24-well plate, we examined a lower initial cell concentration (6.6 × 10^4^ cells/mL) to examine if this has a detrimental effect on the cell growth pattern. At the same time, we compared two conditions: batch culture versus a feeding approach, where half of the conditioned medium (0.5 mL) was removed and replaced by fresh medium every other day. The growth curves for both conditions were very similar and also reached a concentration of 10^6^ cells/mL in five days, indicating that feeding and the initial concentration (10^5^ cells/mL or 6.6 × 10^4^ cells/mL) of the inoculum did not affect the cell growth pattern (Fig. 3C). Furthermore, cells cultured in serum free-media continue to express ILC2-signature-markers after two weeks in culture (Fig. 3D). Overall, our results show that serum-free medium greatly increased the mouse ILC2 expansion compared to culture in RPMI/FBS. In addition, we have cultured and propagated nonactivated, naïve lung ILC2s directly after dissection and purification as described in Fig. 1. Finally, this procedure works equally well for activated and non-activated or naïve ILC2s isolated from tumours (not shown).

## Discussion

In this study we identify conditions to grow and expand Type 2 innate lymphoid cells in tissue culture. This has significant impact for studying ILC2s and for facilitating further downstream applications of ILC2s. We also describe an improved methodology to maximize yields to harvest and isolate lung ILC2s from female mice. This is significant because Kadel and colleagues identified a plausible explanation for this gender bias in murine ILC2 numbers^34^. Due to sex hormone-related factors, female mice appear to accumulate a KLRG1-deficient ILC2 population in the lungs. A lack of this receptor renders ILC2s insensitive to a halt in cell function and therefore more prone to activation and division. Interestingly, in the presence of androgens, these KLRG1-deficient ILC2s are reduced in males. Also, due to differential lung environments, female ILC2s are also more responsive to IL-33 and thus are better type 2 cytokine producers^43^. A second way to improve yields is to pre-treat mice with alarmins before ILC2 isolation. Although the use of IL-7 and TSLP have been reported^44^, IL-33 pre-treatment is a more obvious choice for isolating ILC2s, but there is no consensus on what the recommended dose is. In the simplest approach, the assumption is that the higher the administered dose of IL-33, the higher the yields. Even though IL-33 activates ILC2s, enhancing effector functions and proliferation in the lungs, it also mobilizes gut resident IL-25R^+^ ST2^lo^ ILC2s cells^41,45^ and egresses differentiated ILC2s from the bone marrow to the lung^14^. Despite the efficacy of this pre-treatment, caution is recommended however, as it may lead to the accumulation of functionally heterogeneous ILC2s that can interfere with certain studies.

Although isolation protocols for mouse ILC2s are already well established, we recommend a series of optimizations that can accelerate the process and improve cell viability. During dissection, several protocols recommend a lung perfusion step in ice-cold PBS to clear as many erythrocytes as possible^31,32^. A digestion step is crucial to fully liberate the ILC2s from the lung tissue, and thus, the labware and selection of enzymes is essential to preserve mouse ILC2 viability and expression of surface markers^37^. We employ a mixture of DNase, collagenase IV and hyaluronidase while shaking the lung homogenate at 200 rpm at 37 °C. Interestingly, Duerr & Fritz noticed that using different enzymes like liberase, dispase II or a lung disassociation kit may yield more cells and preserve surface markers^32^.

Whether researchers use large groups of mice to pool ILC2s or smaller IL-33 pre-treated groups, the final single cell suspension will likely take several hours of fluorescent activated cell sorting (FACS) that can affect recovery and viability^46^. To shorten this time, we chose a mouse ILC2 Enrichment Kit (Stemcell Technologies), which treats the lung homogenate with a cocktail of different antibodies. The list of the flow cytometry antibodies has to be considered while planning future in vitro or in vivo immunological studies on isolated ILC2 cells, as different ILC2-related products are available on the market, and some antibodies may affect downstream assays.

Our group has opted for StemSpan serum-free medium for effective murine ILC2 expansion. Our motivation to optimize ILC2 culture came from previous studies, where it has been reported that ILC2s are able to have a positive or negative effect on cancer progression. Studies suggest ILC2 cells contributing to immune evasion by inhibiting NK cell activation in bladder cancer, lung cancers, colorectal cancers , leukemias by promoting myeloid-derived suppressor cell function^47–50^. Conversely, we previously demonstrated that ILC2s play a role in enhancement of pro-inflammatory anti-tumour cytolytic responses, presenting unconventional cells in the immune surveillance process that may have potential to be exploited as a novel cancer immunotherapy^31,51^, and in pancreatic cancers ILC2 cells can prime anti-tumor responses in response to immune checkpoint blockade^52^. Interestingly, during the course of our work, StemSpan has launched two new serum-free media, as well as xeno-free versions, that would better align with any cell-based GMP-compliant therapy. However, despite lacking evidence with serum-free, xeno-free medium specifically for innate lymphoid cell culture, the company reports that the serum-free medium appears to better at propogating cells and results in higher viability compared to other xeno-free versions in terms of human hematopoietic and progenitor cell expansion^53^.

Lymphocytes are non-adherent cells and can be generally grown in flat or round bottomed wells, however with ILC2s, as the yields are generally low, these tend to be cultured in U-bottom 96-well plates to increase cell proximity^24,31,36^. There is no published evidence regarding ILC2 culture mechanics and the advantages of keeping cells in close contact, but our group has explored the effects of clump pipette trituration (and the absence of it) in culture expansion. We have not observed any significant differences nor trends in population dynamics when we consistently apply trituration to keep ILC2s apart (data not included). Although we have not achieved high culture confluency with RPMI/FBS supplemented with IL-33, TSLP, IL-2 and IL-7, other studies demonstrate that by supplementing with IL-2 and different cytokine combinations and an automated tissue dissociation kit, higher densities may be achievable^54^. We isolated nonactivated, naïve resident ILC2s from the lung and their phenotype was identical to those isolated with a procedure that used the aforementioned surface markers. However, we cannot exclude, that in the case of activated ILC2, that some of these recently migrated to the lung.

Our average doubling time for ILC2 is 21 h (h) in serum-free medium, whereas in in RPMI/FBS these cells have a doubling time almost 70 h. For reference, laboratory grown *Escherichia coli* have a doubling time of 19.5 min (min) (ranges between 15 and 20 min), for which they need a significantly higher growth constant of 2.13 h^−1^^55^. The murine tumour cell line B16 divides at a rate of 24.9 h per cell division, slightly slower than ILC2s, while IL-2–sensitive, co-stimulated CD8^+^ T-cells have an impressive doubling time of 5.3 h^56^, essential to mount effective cellular responses against tumours or pathogens. As type 2 cytokine secretors that were recently discovered to function in T_H_1 CD8^+^ T-cell activation^31^, ILC2s do not appear to have the requirement to replicate at such rates.

We demonstrate that it is possible to obtain viable ILC2 sub-cultures by re-seeding on days 6 and 7, where the cell concentration increases steadily (see log curve) but quickly deteriorates at the same time as the primary explant (Fig. 3A). Cell quality is reflected in Fig. 3B of an ELISA assay we performed (not included), where we can observe that the amounts of IL-5 and IL-13 secreted per cell increments with time, as the cell concentration in culture increases. Furthermore, the IL-13 secreted per ILC2 cell belonging to an initial concentration of 194 K cells/mL in a higher dilution (× 1120) reflect the initial contraction phase in culture, probably due to the transfer from an in vivo system to an in vitro one. ILC2 concentration increases more significantly if sub-culturing is carried out early in the exponential phase, on day 1.5, as opposed to days 3, 6 or 7 (data not included). Our data, however, is still aligned with other non-adherent cell culture protocols that recommend sub-culturing during the exponential phase to maximize growth and to prevent genomic instability that can affect the quality of experiments^57,58^. However, as a purified culture, it may be expected that ILC2s produce higher levels of IL5/IL13 however, this was not observed and the reason for this is unclear.

Propagation is possible though the ‘nonactivated’ or ‘naïve’ cells initial numbers are significantly reduced from the IL-33 treated animals. We also observe that starting with a high cell concentration (194 K cells/mL) and a lower one (100 K cells/mL) yields a similar culture growth in the serum-free medium supplemented with IL-33, TSLP, IL-2 and IL-7 (Fig. 3A), meaning that the initial concentration of the inoculum does not have a large impact on expansion. If the initial cell concentration is lower, such as 6.6 × 10^4^ cells/mL (Fig. 3C), we also observe an exponential phase of growth (Fig. 3A). Thus, we find that culturing with low initial ILC2 concentrations is attainable in serum-free medium supplemented with IL-33, TSLP, IL-2 and IL-7, however, the lower limit for a viable expansion remains unknown and has to be determined. Future experiments may further address the characteristics of IL-25 -induced ILC2.

Finally, we compared the effect of two different culture strategies on ILC2s for the course of a week: batch versus feeding (fed-batch). The former involves an absence of medium changes, meaning that the cells will eventually meet a limited supply of nutrients that will cause a decline in viability and cell numbers. The latter is commonly used in large scale bioreactors and involves a continuous supply of one or more nutrients. Technically, our feeding approach cannot be defined as fed-batch because we changed the medium every other day instead of continuously, but ILC2s still have constant access to fresh nutrients. As illustrated in Fig. 3C, in the span of seven days there is no visible difference in culture expansion when comparing these techniques. Our group has used this approach to batch-culture ILC2s for the experiments and have obtained consistent results. This reduces the cost of medium and therefore contributed to economizing experimentation with ILC2s.

## Conclusion

In this study we present an alternative to RPMI 1640 supplemented with FBS to culture murine type 2 innate lymphoid cells. Although serum-free medium was initially recommended for human hematopoietic cells, it has only been tested in one study using human ILC2s, and never with murine ILC2s. Our results demonstrate that culturing mouse, lung ILC2s in serum-free medium supplemented with IL-33, TSLP, IL-2 and IL-7 yields highly viable cells but also a significantly larger expansion than RPMI supplemented with FBS and IL-33, TSLP, IL-2 and IL-7, with the added benefit that there is no need for medium changes over the course of a week. The observed increase in cell concentration was sufficient to allow the culture these ILC2 in wells of larger formats than the 96-well plate, and to sub-culture viable cells from original explants. To fully optimize culture, a cytokine study will be necessary to identify the best combination for ILC2 growth. In addition, we recommend improving freeze–thaw protocols to minimize the number of ILC2 isolations from mice. Given the low numbers of ILC2s yielded by existing methods our studies can improve ILC2 culture and further downstream applications for ILC2s.

## References

[1] Vivier E (2018). Innate lymphoid cells: 10 years on. Cell.

[2] Bernink JH, Germar K, Spits H (2014). The role of ILC2 in pathology of type 2 inflammatory diseases. Curr. Opin. Immunol..

[3] Carriere V (2007). IL-33, the IL-1-like cytokine ligand for ST2 receptor, is a chromatin-associated nuclear factor in vivo. Proc. Natl. Acad. Sci. U. S. A..

[4] Martinez-Gonzalez I, Steer CA, Takei F (2015). Lung ILC2s link innate and adaptive responses in allergic inflammation. Trends Immunol..

[5] Mirchandani AS (2014). Type 2 innate lymphoid cells drive CD4+ Th2 cell responses. J. Immunol..

[6] Gasteiger G, Rudensky AY (2014). Interactions between innate and adaptive lymphocytes. Nat. Rev. Immunol..

[7] Halim TY (2014). Group 2 innate lymphoid cells are critical for the initiation of adaptive T helper 2 cell-mediated allergic lung inflammation. Immunity.

[8] Barlow JL (2012). Innate IL-13-producing nuocytes arise during allergic lung inflammation and contribute to airways hyperreactivity. J. Allergy Clin. Immunol..

[9] Roediger B (2013). Cutaneous immunosurveillance and regulation of inflammation by group 2 innate lymphoid cells. Nat. Immunol..

[10] Moro K (2010). Innate production of T(H)2 cytokines by adipose tissue-associated c-Kit(+)Sca-1(+) lymphoid cells. Nature.

[11] Ikutani M (2012). Identification of innate IL-5-producing cells and their role in lung eosinophil regulation and antitumor immunity. J. Immunol..

[12] Neill DR (2010). Nuocytes represent a new innate effector leukocyte that mediates type-2 immunity. Nature.

[13] Eberl G, Colonna M, Di Santo JP, Mckenzie AN (2015). Innate lymphoid cells. Innate lymphoid cells: a new paradigm in immunology. Science.

[14] Stier MT (2018). IL-33 promotes the egress of group 2 innate lymphoid cells from the bone marrow. J. Exp. Med..

[15] Riedel JH (2017). IL-33-mediated expansion of type 2 innate lymphoid cells protects from progressive glomerulosclerosis. J. Am. Soc. Nephrol..

[16] Oliphant CJ (2014). MHCII-mediated dialog between group 2 innate lymphoid cells and CD4(+) T cells potentiates type 2 immunity and promotes parasitic helminth expulsion. Immunity.

[17] Licona-Limon P, Kim LK, Palm NW, Flavell RA (2013). TH2, allergy and group 2 innate lymphoid cells. Nat. Immunol..

[18] Wong SH (2012). Transcription factor RORalpha is critical for nuocyte development. Nat. Immunol..

[19] Krabbendam L, Bal SM, Spits H, Golebski K (2018). New insights into the function, development, and plasticity of type 2 innate lymphoid cells. Immunol. Rev..

[20] Walker JA, Barlow JL, McKenzie AN (2013). Innate lymphoid cells—How did we miss them?. Nat. Rev. Immunol..

[21] Zhang K (2017). Cutting edge: notch signaling promotes the plasticity of group-2 innate lymphoid cells. J. Immunol..

[22] Silver JS (2016). Inflammatory triggers associated with exacerbations of COPD orchestrate plasticity of group 2 innate lymphoid cells in the lungs. Nat. Immunol..

[23] Silver JS (2016). Erratum: Inflammatory triggers associated with exacerbations of COPD orchestrate plasticity of group 2 innate lymphoid cells in the lungs. Nat. Immunol..

[24] Halim TY, Takei F (2014). Isolation and characterization of mouse innate lymphoid cells. Curr Protoc Immunol.

[25] Halim TY, Krauss RH, Sun AC, Takei F (2012). Lung natural helper cells are a critical source of Th2 cell-type cytokines in protease allergen-induced airway inflammation. Immunity.

[26] Kabata H (2013). Thymic stromal lymphopoietin induces corticosteroid resistance in natural helper cells during airway inflammation. Nat. Commun..

[27] Motomura Y (2014). Basophil-derived interleukin-4 controls the function of natural helper cells, a member of ILC2s, in lung inflammation. Immunity.

[28] Hoyler T (2012). The transcription factor GATA-3 controls cell fate and maintenance of type 2 innate lymphoid cells. Immunity.

[29] Furusawa J (2013). Critical role of p38 and GATA3 in natural helper cell function. J. Immunol..

[30] Mjosberg J (2012). The transcription factor GATA3 is essential for the function of human type 2 innate lymphoid cells. Immunity.

[31] Saranchova I (2018). Type 2 innate lymphocytes actuate immunity against tumours and limit cancer metastasis. Sci. Rep..

[32] Duerr CU, Fritz JH (2017). Isolation of group 2 innate lymphoid cells from mouse lungs. Methods Mol. Biol..

[33] Drake LY, Kita H (2014). Group 2 innate lymphoid cells in the lung. Adv. Immunol..

[34] Kadel S (2018). A major population of functional KLRG1(-) ILC2s in female lungs contributes to a sex bias in ILC2 numbers. Immunohorizons.

[35] Bruce DW (2017). Type 2 innate lymphoid cells treat and prevent acute gastrointestinal graft-versus-host disease. J. Clin. Invest..

[36] Camelo A (2017). IL-33, IL-25, and TSLP induce a distinct phenotypic and activation profile in human type 2 innate lymphoid cells. Blood Adv..

[37] Moro K, Ealey KN, Kabata H, Koyasu S (2015). Isolation and analysis of group 2 innate lymphoid cells in mice. Nat. Protoc..

[38] Karnieli O (2017). A consensus introduction to serum replacements and serum-free media for cellular therapies. Cytotherapy.

[39] Karnieli O (2018). Corrigendum to “A consensus introduction to serum replacements and serum-free media for cellular therapies” [Cytotherapy 19 (2017) 155–169]. Cytotherapy.

[40] Brindley DA (2012). Peak serum: implications of serum supply for cell therapy manufacturing. Regen. Med..

[41] Martinez-Gonzalez I, Matha L, Steer CA, Takei F (2017). Immunological memory of group 2 innate lymphoid cells. Trends Immunol..

[42] Percie du Sert N (2020). Reporting animal research: explanation and elaboration for the ARRIVE guidelines 20. PLoS Biol.

[43] Matha L (2019). Female and male mouse lung group 2 innate lymphoid cells differ in gene expression profiles and cytokine production. PLoS ONE.

[44] Ricardo-Gonzalez RR (2018). Tissue signals imprint ILC2 identity with anticipatory function. Nat. Immunol..

[45] Martinez-Gonzalez I (2018). ILC2 memory: recollection of previous activation. Immunol. Rev..

[46] Fitzgerald DA (2001). Cell sorting: an enriching experience. Sci..

[47] Wang S (2020). Correction: transdifferentiation of tumor infiltrating innate lymphoid cells during progression of colorectal cancer. Cell Res..

[48] Trabanelli S (2017). Tumour-derived PGD2 and NKp30-B7H6 engagement drives an immunosuppressive ILC2-MDSC axis. Nat. Commun..

[49] Schuijs MJ (2020). ILC2-driven innate immune checkpoint mechanism antagonizes NK cell antimetastatic function in the lung. Nat. Immunol..

[50] Wang S (2020). Transdifferentiation of tumor infiltrating innate lymphoid cells during progression of colorectal cancer. Cell Res..

[51] Saranchova I (2016). Discovery of a metastatic immune escape mechanism initiated by the loss of expression of the tumour biomarker interleukin-33. Sci. Rep..

[52] Moral JA (2020). ILC2s amplify PD-1 blockade by activating tissue-specific cancer immunity. Nature.

[53] StemCell_Technologies. Hematopoietic stem and progenitor cells: products for your research. (2020). https://cdn.stemcell.com/media/files/brochure/BR29054-Hematopoeitic_Stem_and_Progenitor_Cells.pdf?_ga=2.215630226.428577042.1613771274-379058216.1613771274.

[54] Moro, K. ILC2 isolation from different mouse tissues. (2019). https://www.miltenyibiotec.com/_Resources/Persistent/d6784274c7c95193d64215ef4bb316df063c3685/ILC2%20isolation%20from%20different%20mouse%20tissues.pdf.

[55] Marr AG (1991). Growth rate of *Escherichia coli*. Microbiol. Rev..

[56] Hwang LN, Yu Z, Palmer DC, Restifo NP (2006). The in vivo expansion rate of properly stimulated transferred CD8+ T cells exceeds that of an aggressively growing mouse tumor. Cancer Res..

[57] Oh JH (2013). Genotype instability during long-term subculture of lymphoblastoid cell lines. J. Hum. Genet..

[58] Millipore_Sigma-Aldrich. *Cell Culture Protocol 5: Subculture of Suspension Cell Lines [Online].* https://www.sigmaaldrich.com/technical-documents/protocols/biology/subculture-of-suspension.html (2020).

